# A concept analysis of the term migrant women in the context of pregnancy

**DOI:** 10.1111/ijn.12600

**Published:** 2017-10-20

**Authors:** Marie‐Clare Balaam, Melanie Haith‐Cooper, Alena Pařízková, Marina Joanna Weckend, Valerie Fleming, Triin Roosalu, Sanja Špoljar Vržina

**Affiliations:** ^1^ University of Central Lancashire Preston UK; ^2^ Faculty of Health Studies University of Bradford Bradford West Yorks UK; ^3^ Department of Sociology, Faculty of Philosophy and Arts University of West Bohemia in Pilsen Plzen Czech Republic; ^4^ Midwifery Research and Education Unit Hannover Medical School Hannover Germany; ^5^ Liverpool John Moore's University Liverpool UK; ^6^ Sociology & Senior Researcher at the Institute of International and Social Studies, School of Governance, Law and Society Tallinn University Tallinn Estonia; ^7^ Department of Political Science Hobart and William Smith Colleges Geneva NY USA; ^8^ Anthropology Institute of Social Sciences Ivo Pilar Zagreb Croatia

**Keywords:** concept, midwifery, migrant, nursing, pregnant, women

## Abstract

**Aim:**

This paper explores the concept of migrant women as used in European healthcare literature in context of pregnancy to provide a clearer understanding of the concept for use in research and service delivery.

**Methods:**

Walker and Avant's method of concept analysis.

**Results:**

The literature demonstrates ambiguity around the concept; most papers do not provide an explicit or detailed definition of the concept. They include the basic idea that women have moved from an identifiable region/country to the country in which the research is undertaken but fail to acknowledge adequately the heterogeneity of migrant women. The paper provides a definition of the concept as a descriptive theory and argues that research must include a clear definition of the migrant specific demographics of the women. This should include country/region of origin and host, status within the legal system of host country, type of migration experience, and length of residence.

**Conclusion:**

There is a need for a more systematic conceptualization of the idea of migrant women within European literature related to pregnancy experiences and outcomes to reflect the heterogeneity of this concept. To this end, the schema suggested in this paper should be adopted in future research.

## SUMMARY STATEMENT

What is already known about this topic?

- There is an increasing concern with the health of pregnant migrant women in Europe.
- There is a lack of clear definition of what is meant by the concept migrant women in European literature focusing on pregnancy experience and outcomes.
- This ambiguity negatively effects the comparability and so utility of research on this topic.

What this paper adds?

- An analysis of the use of the concept migrant women in contemporary European health and social care literature focusing on pregnancy experience and outcomes.
- A descriptive theory, which provides the basis for a more nuanced conceptualization of this concept.
- A schema based on 4 descriptive aspects surrounding pregnant migrant women, which could provide a useful framework for further empirical research.

The implications of this paper:

- The implementation of the theory presented would provide a more nuanced basis for research acknowledging the heterogeneity of migrant experience.
- A clearer definition of the characteristics of participants in future studies would improve the comparability of research.
- The schema offers a practical tool, which could be adopted by future researchers and/or policymakers.

## 1 INTRODUCTION

The United Nation's (UN) definition of international migrant is “a person who is living in a country other than his or her country of birth” (UN, 2016, p. 4), with an estimation of over 244 million international migrants in 2015. Europe has seen a significant increase in the numbers of international migrants (76 million) (UN, 2016) due to the ongoing crisis in Syria with over 1 million refugees arriving in Europe in 2015 (Eurostat, 2016).

A growing body of research demonstrates that many migrants experience poorer health than nonmigrant populations (World Health Organisation (WHO), 2015). Worldwide, women make up 48% of international migrants (UN, 2016), and with a median age of 29 to 43, these include large numbers of women of childbearing age (OECD, 2013). The reproductive health of these women is of increasing concern to researchers, practitioners, and policymakers (du Monde, 2014; International Women's Health Coalition, 2013). While pregnancy and birth are significant life and health events for all women, research demonstrates that for many migrant women, the perinatal period is one that is particularly challenging (Song, Ahn, Kim, & Roh, 2016; UNHCR, 2015), and the outcome of which can influence the health in later life of both mother and infant (Rutayisire et al., 2016). International research suggests that many migrant women struggle to access optimal maternity care and experience poorer pregnancy outcomes than nonmigrant women (Carolan, 2010; Essen, Hanson, Ostergren, Lindquist, & Gudmundsson, 2000; Gagnon, Zimbeck, & Zeitlin, 2009). However, inconsistent definitions of migrant women has led to difficulties in gaining insight into the reasons for these poorer outcomes. This lack of specificity in the use of terminology can lead to a failure to differentiate between the maternity care needs and experiences of different groups of migrant women (Gagnon et al., 2009; Viken, Balaam, & Lyberg, 2017) and make the comparability and interpretation of such research data problematic.

Understanding the heterogeneity of migrant women and their experiences is essential when providing maternity care because their different experiences and situations may affect their care needs. For example, pregnant women who have been forced to migrate, including asylum seekers and refugees, may have experienced war and sexual violence, which may have had an impact upon their physical and mental health (Aspinall & Watters, 2010), meaning they have different needs from women who migrated voluntarily, for example, economic migrants. Consequently, further consideration needs to be given to the concept migrant women, ensuring that it is clearly defined to include the range of migrant experiences both forced and voluntary. This will inform health providers of the potential backgrounds of migrant women accessing maternity care and help to tailor maternity care to the individual needs of women and families.

## 2 REVIEW METHODS

### 2.1 Aims

The aim of this paper is to explore the concept of migrant women as used within contemporary academic literature on maternity to provide a clearer understanding of the concept within the context of pregnancy and to propose a clear operational definition of the term for use in research, policy, and targeted health service delivery. This will allow greater clarity in and more appropriate comparability between research. This focus on maternity reflects the academic literature, which demonstrates the significance of this period for the health of migrant women. This paper focuses on European research, acknowledging the current increase in migration in Europe.

### 2.2 Design

This paper provides a theoretical concept analysis focusing on peer reviewed articles (following Risjord, 2009) to highlight the concept as it appears in scientific literature and to acknowledge the importance of this literature in the creation of *authoritative knowledge* and practice (Risjord, 2009). This method was selected as it aims to “create conceptual and terminological clarity” (Nuopponen, 2010, p. 6) and “can provide a knowledge base for practice by offering clarity and enabling understanding” (Baldwin, 2008 p. 50). This concept analysis uses the approach developed by Walker and Avant (2011). This method follows an 8 step procedure, which includes identifying the concept, aims, and purpose of the analysis, establishing all uses of the concept, determining the defining attributes of the concept, constructing cases to further clarify the concept, identifying antecedents and consequences, and finally, where appropriate, defining empirical referents.

The first 2 steps, concept selection and determining the aims of analysis, have been described above. The rest of the steps of starting with the identification of all uses of the concept are detailed below.

### 2.3 Search methods

We consider 6 databases as the most relevant to maternity care and migration across a range of disciplines: Scopus, ASSIA, Sage, Medline, Psych articles, and Pubmed. Between September and November 2015, an electronic search using the keywords *pregnant*, *migrant*, and *women* was undertaken of articles published between 2005 and 2015. As the terms *immigrant* and *migrant* were used in searched literature interchangeably, the search includes both of this variations. The search identified 1387 articles (Table 1). In the next step, the duplicates were removed and the initial exclusion criteria were applied when reviewing abstracts of all articles.

**Table 1:** Database search results

Database	Number of Initial Hits	Number After Initial Exclusions
Scopus	259	72
ASSIA	14	7
Sage	1001	58
Medline	6	0
Psych articles	7	0
Pubmed	100	1

The initial exclusion criteria for articles were the following:

- No mention of keywords in abstract;
- Not written in English;
- Not focused on migration to a European country;
- Historical articles, books, letters;
- Publication before 2005

The remaining 138 articles were reviewed in full text, each reviewed by 2 authors of this paper. If there was a disagreement, the 2 authors discussed this and came to a resolution. The process was performed in 2 steps. In the first stage of the reviewing process, 115 articles were excluded as they

- did not pertain to the European setting (n = 6);
- did not have maternity/pregnancy and migrant women as the primary focus of the paper (n = 104). This included articles, which used pregnant migrant women solely as a risk group in articles whose central focus was the exploration of a pathological condition.
- are not available in full text (n = 5).

**Figure 1:**
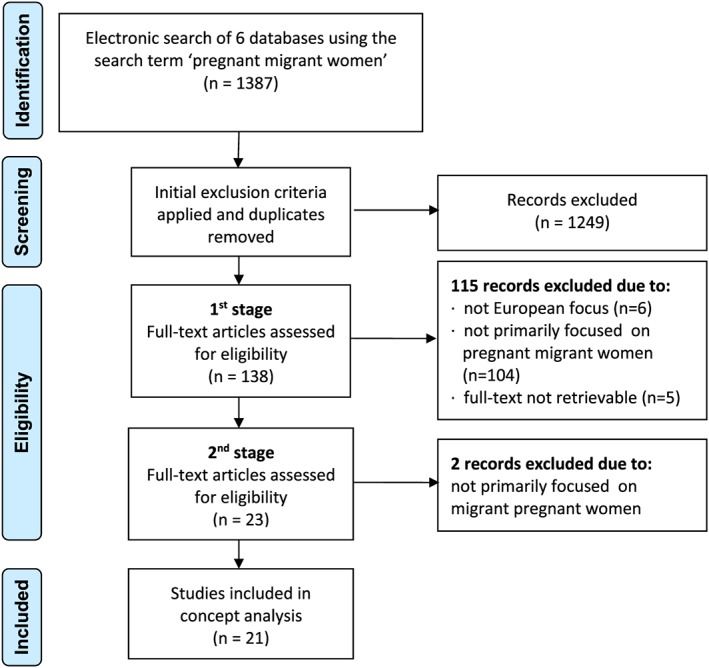
Flow diagram of included studies

In the second stage, remaining 23 articles were again divided between the authors for the second stage of the reviewing process. Two more articles were rejected at this point for not fully meeting the criteria of having pregnant migrant women as their primary focus. The outcome of the selection process was 21 articles available for concept analysis (Figure 1).

### 2.4 Quality appraisal

No formal method of quality appraisal was used in this study as it was important to include a wide range of published literature to explore the full range of ways in which the concept under analysis is used in maternity literature. All articles were reviewed by the authors of this analysis, which guaranteed that rigour and authors' cross European and interdisciplinary backgrounds added depth to the process. Conflicts were resolved by consensus, and if no consensus was reached, a third reviewer was consulted.

### 2.5 Data abstraction

The 21 included articles were reviewed with each article being summarized in a database, identifying how the authors defined and used the concept of migrant women and the key focus of the article (Table 2).

**Table 2:** Data abstraction

			Included in Criteria of Definition						
Article	Focus of Paper/Definition	Terms Used	Region/Country of Birth	Nationality	Migration Status	Cause of Migration	Length of Stay or Generation	Ethnicity	
Almeida et al. (2014)	Migrant women in Portugal	Im/migrant women, immigrants,	•		•				
Balaam et al. (2013)	Migrant women. Literature review.	Migrant women as wide category including: refugees, asylum seekers, illegal migrants, economic migrant, transient			•	•			
Binder et al. (2012)	Immigrant African women from sub‐Saharan Africa	Immigrants, immigrant African women	•					•	
Bray et al. (2010)	A8 migrants (2004 EU Accession countries)	New European migrants, A8 migrant population	•		•				
Carolan (2010)	Sub‐Saharan refugee women who have resettled in developed countries. Review of the literature.	Sub‐Saharan refugee women, im/migrant women, immigrants	•		•	•			
David et al. (2006)	“Women with a migrant ethnic background was narrowed down to the largest group of migrants in Germany, namely those of Turkish ethnicity” (p. 272)	Immigrants from Turkey, pregnant migrants, non‐German ethnicity, mothers of non‐German ethnicity, migrant, German women	•					•	
Karl‐Trummer et al. (2006)	Migrant/ethnic minority women	Migrant/ethnic minority, migrant women						•	
Kilner, H. (2014).	Opinion paper focused on migrants and immigration legislation in UK	Migrants, international migrants			•				
Mantovani, N., & Thomas, H. (2014).	Young, Black women, looked after by the state in the UK, of this group the majority are “migrants or asylum seekers”	Migrants and asylum seekers			•			•	
Merten et al. (2007)	Migrants in Switzerland	Migrants, non‐Swiss nationality, non‐Swiss mothers, mothers of foreign nationality	•	•					
Munro et al. (2012)	Undocumented pregnant migrants. “We define migrants as people who, for a variety of reasons choose to leave their home countries & establish themselves either permanently or temporarily in another country” (p. 281). Literature review.	Undocumented pregnant women, undocumented migrants, uninsured migrants, refugee claimants			•				
Perez Ramirez et al. (2013)	Pregnant immigrant women in Spain	Pregnant immigrant women in Spain							
Ramos et al. (2011)	Immigrant women in Spain	Immigrant vs native, foreign people, migrant pregnant women, migrants vs Spanish group	•						
Reeske et al. (2011)	Women from different regions of origin	Maternal migrant background, women from different regions of origin, women from Germany or women with/without migrant backgrounds	•						
Tariq et al. (2012)	Pregnancy of African women living with HIV in the UK. “African was defined as being of Black ethnicity and having been born in sub‐Saharan Africa. Women of mixed, white or Asian ethnicities who were born in sub‐Saharan African were not defined as African” (p. 2)	Pregnant African women living in the UK	•				•	•	
Vaiou and Stratigaki (2008)	Albanian women settled in Athens	Women migrants in Athens, (Albanian) migrant women	•	•		•			
Velemínský et al. (2014)	Immigrants in the Czech Republic from Vietnam, Mongolia, and Ukraine	Immigrants, foreigners, national minorities	•						
Wolff, Epiney, et al. (2008)	Undocumented migrants in Geneva	Undocumented migrants/women			•				
Wolff, H., Lourenço, A., et al. (2008).	Undocumented migrants in Switzerland	Undocumented migrants, women with legal residency permit			•				
Wolff, H., Stalder, H., Epiney, M., Walder, A., Irion, O., & Morabia, A. (2005).	Undocumented pregnant immigrants in Geneva	Undocumented pregnant immigrants in Geneva, undocumented, uninsured immigrants			•				
Yeasmin and Regmi (2013)	“Pregnant British Bangladeshi women had lived in the UK for at least 10 years (they were literally considered as the first generation of such immigrants)” (p. 410)	Pregnant British Bangladeshi women, migrant British Bangladeshi women,	•				•	•	

### 2.6 Data analysis

Analysis was undertaken using the concept analysis method developed by Walker and Avant (2011). Following this multistage method, the defining attributes, which described the basic concept, were then identified. To clarify the meaning of the concept further, model‐related and contrary cases were identified. Finally, antecedents and consequences of the concept were explored and described.

## 3 RESULTS

### 3.1 Definition

The concept explored in this paper is that of migrant women within the context of pregnancy. A basic definition of migration is “the movement of a person or people from one country, locality, place of residence, etc., to settle in another” (OED, n.d.). Alongside this, migrants are the actors; they are the entity *that migrates* or that is *characterized by migration* (OED). The definition of migrant is often linked to the concept of migration, but these are 2 distinct terms. Migration can be seen as a process and migrants as actors in a particular context.

### 3.2 Concept as used in the literature

The 21 included articles covered a broad European perspective; an explicitly cross national perspective (n = 3), United Kingdom (n = 6), Switzerland (n = 4), Germany (n = 2), Spain (n = 2), Portugal (n = 1), Greece (n = 1) Czech Republic (n = 1), and Austria (n = 1). The articles come from a range of disciplines including midwifery, maternity care, public health, reproductive health, and sociology. They use a range of methodological approaches, addressing a variety of relevant issues, most commonly access to and use of maternity care by migrant women (Kilner, 2014; Munro, Jarvis, Munoz, D'Souza, & Graves, 2012; Binder, Johnsdotter, & Essén, 2012; Wolff, Epiney, et al., 2008; Bray et al., 2010; Karl‐Trummer, Krajic, Novak‐Zezula, & Pelikan, 2006). However other issues include maternal and infant outcomes for migrant women (David, Pachaly, & Vetter, 2006; Merten, Wyss, & Ackermann‐Liebrich, 2007; Perez Ramirez, Garcia‐Garcia, & Peralta‐Ramirez, 2013; Reeske, Kutschmann, Razum, & Spallek, 2011); migrant women's experiences of perinatal care in their host country (Almeida, Casanova, Caldas, Ayres‐de‐Campos, & Dias, 2014; Balaam et al., 2013; Velemínský et al., 2014); reproductive health including HIV, Chlamydia, and Toxoplasmosis (Tariq, Pillen, Tookey, Brown, & Elford, 2012; Wolff, Epiney, et al., 2008; Ramos et al., 2011); the health status of migrant women (Carolan, 2010; Wolff et al., 2005); decision‐making in pregnancy (Mantovani & Thomas, 2014); and identity and settlement (Vaiou & Stratigaki, 2008).

Only 4 texts explicitly defined pregnant migrant women (Balaam et al., 2013; Binder et al., 2012; Carolan, 2010; Munro et al., 2012). The remaining 17 articles offered differing levels of definition and complexity of conceptualization. There was commonly a lack of detailed engagement with the identity of these women, beyond that they had arrived in the country where the research was taking place, for example, “30 immigrant women” (Perez Ramirez et al., 2013), “during the study period, the hospital provided medical care to 290,481 inhabitants, from three municipalities ... a total of 44,341 of them were foreign people” (Ramos et al., 2011 p. 1448). This approach presents pregnant migrant women as a homogenous group, commonly in opposition to an equally homogenous nonmigrant population. In some papers, a more detailed engagement with the concept takes place, in the demographics section rather than in the initial research design, commonly leading to a situation in which the complexity and heterogeneity of the concept emerge only partially and/or very late in the presentation of the research (Perez Ramirez et al., 2013; Almeida et al., 2014).

The breadth of the term migrant, and its lack of specificity, is demonstrated in the wide range of terms used interchangeably to refer to migrant women. These include im/migrant women/mother (Balaam et al., 2013; Perez Ramirez et al., 2013), undocumented pregnant women (Munro et al., 2012), young Black teenage mothers (Mantovani & Thomas, 2014), women with and without migrant background (Reeske et al., 2011), documented and irregular migrants (Kilner, 2014), undocumented migrants (Wolff, Lourenço, et al., 2008; Wolff, Epiney, et al., 2008), refugee (Carolan, 2010), asylum seekers (Mantovani & Thomas, 2014), ethnic minority group (Karl‐Trummer et al., 2006; Mantovani & Thomas, 2014), educational migrants (Mantovani & Thomas, 2014), women from foreign region (Reeske et al., 2011). The interchangeability of terms suggests a lack of clarity in the use of the concept. Similarly, the terms ethnic minority and migrant are not clearly differentiated within some of the literature (David et al., 2006; Karl‐Trummer et al., 2006; Tariq et al., 2012).

### 3.3 Defining attributes

The defining attributes of a concept are those characteristics, which are consistently associated with the concept and that act to differentiate it from other similar or related ones (Walker & Avant, 2011). In the reviewed literature, 3 defining attributes were identified for the concept of migrant women in the context of pregnancy. This first one is that of being a woman, the second that women (or their parents or grandparents) have moved to the country in which the research is being undertaken, and the third that these persons have moved from an identifiable country or region of origin/birth. In addition, the location of women within the legal structures of the host country has been included as an attribute. This was commonly, although not comprehensively, used within the literature and, when used, had an important impact on the understanding of the concept. All of these attributes appear within the context of pregnancy.

#### 3.3.1 Movement to the country in which the research is undertaken

Common to all articles is the idea that migrant women (or their parents/grandparents) have moved to the country in which the research has been undertaken. The women are referred to as migrant, immigrant, or international migrant, often interchangeably, “30 immigrant women” (Perez Ramirez et al., 2013, p. 350), “migrant women in Geneva” (Wolff et al., 2005, p. 1250), “a group of immigrants in a large urban area in northern Portugal” (Almeida et al., 2014, p. 720).

#### 3.3.2 Movement from an identifiable country or region of origin/birth

All articles expand this initial idea to include an identification of the women (or parents/grandparents) having moved from an identifiable country or region to the host country. Migrants are characterized by the fact that they have a different and specifically identified country of origin to the county they are currently residing in. In one article, this is expressed in a very broad and oppositional sense as “women from different regions of origin compared to women from Germany” (Reeske et al., 2011, p. 2). Other research clusters countries of origin into broader geographical areas or regions; eg, “54 immigrant African women from sub Saharan Africa” (Binder et al., 2012, p. 2030), “Migrants from A8 countries” (Binder et al., 2012; Bray, Gorman, Dundas, & Sim, 2010; Merten et al., 2007; Munro et al., 2012; Vaiou & Stratigaki, 2008; Wolff et al., 2005; Wolff, Lourenço, et al., 2008). In other articles, the country of birth (Ramos et al., 2011; Almeida et al., 2014; Bray et al., 2010), region of birth (Tariq et al., 2012), or nationality (Reeske et al., 2011; Wolff, Lourenço, et al., 2008) is more specifically identified. One article develops the idea of place of origin further by using an additional economic category, making a distinction between high‐income and low‐income countries (Binder et al., 2012).

#### 3.3.3 Women's position in the host country's legal system

Thirteen of the 21 papers include in their conceptualization some exploration of the differing positions women may occupy as *migrant* within the legislative and administrative system of the country in which the research is undertaken. In some cases, this was very broad, acknowledging that there are a range of positions women can occupy. For example, refugees, asylum seekers, illegal migrants, economic, migrant, and transient (Balaam et al., 2013). Others are less generalized in their terminology but still use the terms refugees and asylum seekers (Balaam et al., 2013; Kilner, 2014; Mantovani & Thomas, 2014; Tariq et al., 2012) in an undifferentiated way.

Other work identifies particular statuses that migrant women may embody, for example, regular (Kilner, 2014; Perez Ramirez et al., 2013). Regular migrants have “correct documentation” and travel “though legal channels” (Kilner, 2014, p. e590) or are “legally admitted” and “legally authorized to reside” (Perez Ramirez et al., 2013, p. 349). Irregular migrants are the opposite. They are not legally admitted to the host country; they could have “fail[ed] to renew their immigration license” (Perez Ramirez et al., 2013, p. 349), overstayed their visa, or are victims of human trafficking (Kilner, 2014). Other work considers the idea of secure and insecure status; “Secure immigration status is defined as being a UK citizen, a recognized refugee or having exceptional or indefinite leave to remain. Anyone not in these categories is defined as having insecure immigration status” (Tariq et al., 2012, p. 6), as well as documented and undocumented migrants (Wolff et al., 2005; Wolff, Epiney, et al., 2008). Other articles acknowledge, but rarely consider in any depth, that migrant women can be “economic migrants” (Balaam et al., 2013; Vaiou & Stratigaki, 2008; Wolff et al., 2005) and “educational migrants” (Mantovani & Thomas, 2014), “undocumented … uninsured migrants and refugee claimants” (Munro et al., 2012) and “A8 migrant population” (Bray et al., 2010). These articles provide a more complex concept of migrant women and being to challenge the homogeneity assumed in the articles, which rely solely on one of the basic attributes identified earlier and as such move beyond the generalization of migrant women and begin to differentiate between migrant women.

### 3.4 Model, contrary, and related cases

A model case selected from the literature reviewed, which fitted Walker and Avant's (2011, p. 169) idea of providing a *paradigmatic example*, is that of women, who moved from countries in sub‐Saharan regions in Africa, including Somalia, Ghana, Nigeria, Senegal, and Eritrea, to the United Kingdom. They were currently resident in the United Kingdom (length of residence varied between 1 and 20 years) and had received/were receiving maternity‐related care in the United Kingdom (Binder et al., 2012).

A contrary case is one where there is an absence of the key defining attributes previously identified. This would be individuals who were not women and had not moved from their country of origin to a different country, as this is a situation in which none of the defining concepts are present.

A related case, (Walker & Avant, 2011, p. 171) which is “related to the concept being studied” but does not “contain all the defining attributes,” would be that of women who have undertaken migration with in country boundaries and, thus, would include women accessing maternity care in China who may have migrated long distances but not crossed a national border (Shaokang, Zhenwei, & Blas, 2002).

### 3.5 Antecedents

Antecedents are described as “events or incidents that must occur or be in place prior to the occurrence of the concept” (Walker & Avant, 2011, p. 173) In this case, there are 4 antecedents. Firstly, the woman has to be pregnant, as this is the context in which the concept is located for this study. Secondly, the presence of the historical, geopolitical concepts of nation states, nationality, and internationally recognized boundaries. The existence of these concepts mean people can then move from one region where they are deemed to originate to one in which they are deemed (certainly initially) not belong to or originate from. The third antecedent is the action to leave the country of origin and move to a different country. This decision can be determined by a range of situations and motivations including “populations displaced as a result of war/and or famine” (Carolan, 2010, p. 407), seeking refuge or asylum (Balaam et al., 2013; Kilner, 2014; Mantovani & Thomas, 2014; Munro et al., 2012), as well as voluntary motives including economic conditions (Balaam et al., 2013; Munro et al., 2012; Wolff et al., 2005), education (Mantovani & Thomas, 2014), and family reunification (Vaiou & Stratigaki, 2008). The fourth antecedent is the physical process and ability to move from/make the journey from one country to another.

### 3.6 Consequences

Consequences as defined by Walker and Avant (2011, p. 173) are “events or incidences that occur as a result of the occurrence of the concept … the outcomes of the concept.” There are 4 key consequences of the concept of migrant women in the context of pregnancy based on the literature reviewed. They are, firstly, that women entering a new country as migrants are located within and subject to a range of socio‐legal‐cultural‐economic discourses and practices different to those applied to women deemed to be native to/nonmigrant (Perez Ramirez et al., 2013; Wolff et al., 2005; Almeida et al., 2014; Carolan, 2010; Kilner, 2014; Wolff, Lourenço, et al., 2008; Wolff, Epiney, et al., 2008); secondly, that these women are forced to seek ways to adapt to their new situation as pregnant women in “the new country” (Balaam et al., 2013, p. 1919; Perez Ramirez et al., 2013, p. 348; Almeida et al., 2014; Yeasmin & Regmi, 2013; Mantovani & Thomas, 2014); and thirdly, that these women will be involved in the healthcare system of their host country because of their pregnancy. This interaction is affected by their movement to the country and their identification as migrants. Evidence from these papers shows that women newly arrived in a country often face a range of challenges in accessing the same level and quality of care than women born in that country (Wolff et al., 2005; Almeida et al., 2014; Carolan, 2010; Kilner, 2014; Velemínský et al., 2014; Mantovani & Thomas, 2014). Finally, newly arrived women commonly have poorer pregnancy outcomes than women born in the host country (Perez Ramirez et al., 2013; Reeske et al., 2011; Carolan, 2010; Karl‐Trummer et al., 2006; Mantovani & Thomas, 2014; David et al., 2006).

## 4 DISCUSSION

The literature reviewed demonstrates an ambiguity around the concept of migrant women within the context of pregnancy. Most papers do not provide an explicit or detailed definition of what they mean by the concept. All the papers do include the most basic idea that women (or their parents or grandparents) have moved from an identifiable region or country to the country in which the research is undertaken. Others seek to add some depth by including an acknowledgement of the differing legal positions women may occupy as a *migrant* within the country in which the research was undertaken, a crucial issue in shaping life chances in the new country (Waters, Pineau, & M. (Eds.)., 2016). They superficially engage with reasons for migration, thus, to some degree, acknowledging the heterogeneity of migrant women. This is critical when considering the different health needs of women in the host country. Some papers discuss nationality and ethnicity; however, these are generally not used in a productive way. They are used primarily as an oppositional category identifying migrants as *the other* in opposition to women born in the host country (Ramos et al., 2011; Merten et al., 2007; Reese et al., 2011; David et al., 2006) or in a way which fails to differentiate ideas of migrant and ethnic or nationality and ethnicity (David et al., 2006; Karl‐Trummer et al., 2006). There is also a lack of clarity over ideas of generation and time spent in the host country with no real analysis of the difference between first and second generation migrant women even though these issues have a significance in women's ability to access healthcare (Merry et al., 2016).

This ambiguity and lack of commonly shared understanding of the concept of pregnant migrant women affect the utility of research by reducing the efficacy of comparative analysis for researchers, policymakers, and practitioners seeking to improve care to for such women. There is a need for a clearer and more systematic conceptualization of the idea of migrant women within European literature on pregnancy experiences and outcomes to reflect the heterogeneity of experience often subsumed by the idea of a migrant woman. We argue that all literature addressing the maternal and perinatal health and/or experiences of migrant women should include a clear definition of the migrant‐specific demographics of the women. This should comprise the following:

- country or region of origin and host;
- status within the legal system of host country;
- type of migration experience (voluntary/ forced);
- length of residence/generation.

### 4.1 Strengths and Limitations

This paper proposes a definition for the concept *migrant women* as a descriptive theory. The study focuses on publications written in English focusing on migration into a European country, so its applicability to a non‐European context may be contested. The multidisciplinary and cross‐European perspective of authors add value to the analysis as it ensures that the concept and its defining attributes have been explored from a number of perspectives.

## 5 CONCLUSION

An increasingly mobile global population means that the ability of European maternity services to meet the needs of, and provide optimal care for, women who have recently migrated to their countries is a significant issue. High‐quality relevant research is crucial for policymakers and practitioners in this area to make informed decisions. This study has identified a gap in existent knowledge in terms of a lack of consistency in categorizing migrant women, which has an impact upon the quality and applicability of literature produced. Building on an analysis of the existing European literature, this study has developed a schema, which we suggest that needs to be used to increase the validity, transferability, and utility of research on pregnant migrant women, which will in turn inform the policies, practices, and education of health professionals in this area. Future work needs to ensure that data collection is nuanced enough to recognize the heterogeneity of contemporary migration. Research can then explore with more clarity the complex issues that affect the interaction of migrant women with the maternity care systems. This work also has implications for health professionals working in this area. Application of the schema this study has developed will help practitioners to more clearly identify and, thus, address needs of migrant women, from whatever background, by providing care that is tailored to their specific needs.

## CONFLICT OF INTEREST STATEMENT

The authors declare no conflict of interest.

## AUTHORSHIP STATEMENT

The authors confirm that all listed authors meet the authorship criteria and that all authors are in agreement with the content of the manuscript.
